# Tuning the Coupling in Single‐Molecule Heterostructures: DNA‐Programmed and Reconfigurable Carbon Nanotube‐Based Nanohybrids

**DOI:** 10.1002/advs.201800596

**Published:** 2018-08-14

**Authors:** Mark Freeley, Antonio Attanzio, Alessandro Cecconello, Giuseppe Amoroso, Pierrick Clement, Gustavo Fernandez, Felice Gesuele, Matteo Palma

**Affiliations:** ^1^ School of Biological and Chemical Sciences Materials Research Institute and Institute of Bioengineering Queen Mary University of London Mile End Road London E1 4NS UK; ^2^ Organisch‐Chemisches Institut Westfälische Wilhelms‐Universität Münster Corrensstrasse 40 48149 Münster Germany; ^3^ Department of Physics University of Naples “Federico II” Via Cintia, 26 Ed. 6 80126 Napoli Italy

**Keywords:** carbon nanotubes, dynamic nanohybrids, single‐molecules, stimuli‐responsive heterostructures

## Abstract

Herein a strategy is presented for the assembly of both static and stimuli‐responsive single‐molecule heterostructures, where the distance and electronic coupling between an individual functional nanomoiety and a carbon nanostructure are tuned via the use of DNA linkers. As proof of concept, the formation of 1:1 nanohybrids is controlled, where single quantum dots (QDs) are tethered to the ends of individual carbon nanotubes (CNTs) in solution with DNA interconnects of different lengths. Photoluminescence investigations—both in solution and at the single‐hybrid level—demonstrate the electronic coupling between the two nanostructures; notably this is observed to progressively scale, with charge transfer becoming the dominant process as the linkers length is reduced. Additionally, stimuli‐responsive CNT‐QD nanohybrids are assembled, where the distance and hence the electronic coupling between an individual CNT and a single QD are dynamically modulated via the addition and removal of potassium (K^+^) cations; the system is further found to be sensitive to K^+^ concentrations from 1 pM to 25 × 10^−3^ m. The level of control demonstrated here in modulating the electronic coupling of reconfigurable single‐molecule heterostructures, comprising an individual functional nanomoiety and a carbon nanoelectrode, is of importance for the development of tunable molecular optoelectronic systems and devices.

## 1 Introduction

The ability to interface individual molecules and nanomoieties to nanoelectronic systems with single‐molecule control is key for the fabrication of next generation molecular (opto)electronic devices.^1^ In particular, it is of uttermost importance to control both the exact position of active molecules or nanostructures onto nanoelectrodes, and the molecule–electrode separation, as small changes in the organization of the individual nanomoieties forming such nanohybrids can have a major impact on the coupling between the different components.^2^ Additionally, the ability to dynamically regulate the coupling in single‐molecule heterostructures represents a powerful tool toward the fabrication of stimuli‐responsive and reconfigurable systems for both optoelectronic and sensing applications. Lastly, a solution‐based approach,^3^ in contrast to top‐down lithographic methodologies,^4^ is highly desirable toward the facile and low‐cost (solution processable) fabrication of nanoscale devices.[[qv: 1b,5]]

In this regard, carbon‐based nanomaterials, such as carbon nanotubes (CNTs) and graphene, have emerged as promising nanoelectrodes and functional scaffolds for nanoelectronic heterostructures, thanks to their excellent charge transport properties and enhanced mechanical characteristics.^6^ Their facile functionalization,[[qv: 6c,7]] also in solution, further makes them ideal for the fabrication of nanohybrids with enhanced optoelectronic features and potential (bio)chemical sensing properties.^8^

Different nanomoieties have been coupled to functional carbon nanostructures, from metal and semiconducting nanoparticles/nanorods^9^ to biological (macro)molecules such as proteins and nucleic acids.[[qv: 2b,10]] Nevertheless, many of the strategies pursued are nonspecific in terms of attachment sites and lack single‐molecule control over the number and distance between the nanomoiety and the carbon nanostructure; this in turn results in an uncontrolled electronic coupling between the nanohybrid components. In particular, even if dimensionality effects have been investigated at the nanoscale^11^ and with single‐molecule resolution,[[qv: 2b,c,12]] challenges remain in precisely controlling both the position and number of nanomoieties per carbon nanoelectrode, at the same time as the nanoscale distance between the two components: essential criteria for the design and implementation of single‐molecule devices.

Here we present a strategy for the controlled formation of reconfigurable single‐molecule heterostructures, where the spacing between a functional nanomoiety and an individual carbon nanostructure is controlled and dynamically tuned by a DNA spacer, employed as a molecular ruler. As a proof of concept, we assembled individual single‐walled CNTs (SWCNTs) coupled to single colloidal semiconductor nanocrystals (Quantum Dots, QDs), chosen as model systems due to their tunable emissions and broad absorbances that further make them ideal candidates for novel light harvesting systems in photovoltaics and light emitting diodes;^13^ A bioinspired approach was pursued via the use of DNA as the linking moiety, due to its demonstrated ability to chemically program the assembly of nanoparticle‐based materials.^14^ In particular, we altered the number of bases in a double stranded (ds)DNA, in order to regulate the nanoscale distance between a SWCNT and a QD, in 1:1 nanohybrids. This in turn allowed us to modulate the coupling between the two nanostructures with single‐molecule control, as demonstrated via static and time‐resolved photoluminescence (PL) investigations as well as single‐molecule measurements: the ability to control the electronic coupling in such heterostructures is an essential attribute for future device implementation. In addition, reversibly reconfigurable heterostructures were assembled where a Guanidine(G)‐rich sequence was used as a linker. The QD's position relative to the end of the SWCNT could then be controlled by the addition and removal of K^+^, which induces the folding of the sequence into a G‐quadruplex (G4) and shortens the distance between the two components^15^ (cryptand 222 allowed us to revert the linker back to its extended conformation restoring the original distance between the two nanostructures). We demonstrate how this stimuli‐responsive strategy allows real‐time control over the coupling between the SWCNT and QD and can be further exploited for the sensing of K^+^ from mM to pM concentrations.

## 2 Nanohybrids Assembly

**Figure 1:**
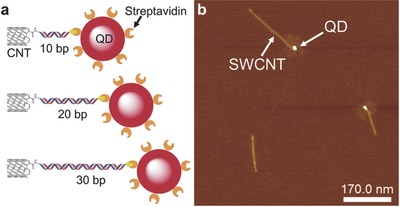
a) Schematics for the SWCNT‐QD nanohybrids with DNA linkers of different length and b) representative AFM image of the SWCNT‐10 bp‐QD heterostructures.

The SWCNTs used in this study underwent a mild acid treatment, followed by dispersion in aqueous solution via DNA wrapping; the SWCNTs were subsequently separated by size exclusion chromatography (see Figure S1, Supporting Information).^16^ The DNA wrapping further protects the side‐wall of the nanotubes, leaving only the terminal ends available for functionalization.[[qv: 2b,12a,17]] A heterobifunctional dsDNA containing a biotin at one end and an amino group at the opposite end was tethered to the carboxylic groups on the SWCNT termini via a simple amidation reaction (see Figure S2, Supporting Information).[[qv: 2b,12a,17a]] This effectively resulted in SWCNTs exhibiting biotin‐terminated dsDNA at their end. Streptavidin‐QD conjugates were then anchored to the SWCNTs biotin termini via biotin‐avidin recognition,^18^ directly in solution: an optimized mass ratio (see Figure S2b, Supporting Information) allowed us to form 1:1 SWCNT‐QD hybrids linked by dsDNA. Notably, by employing duplexes of either 10 base pair (bp), 20 bp, or 30 bp length (see **Table** **1**), we could form nanohybrids where the distance between the QD and the SWCNT is precisely controlled by the employed dsDNA linker (see schematic in **Figure** **1**a).

**Table 1:** Sequences used in each nanohybrid. All sequences are written from 5‐prime to 3‐prime. All modifications are 5‐prime with the exception of **7** which has a 3‐prime modification

Nanohybrid	Oligo no.	Sequence
10 bp	**1**	Amine—CAGGCTCAGG
10 bp	**2**	Biotin—CCTGAGCCTG
20 bp	**3**	Amine—TGCTATGCAGCAGGCTCAGG
20 bp	**4**	Biotin—CCTGAGCCTGCTGCATAGCA
30 bp	**5**	Amine—TGCTATGCAGCGGTCAACTACAGGCTCAGG
30 bp	**6**	Biotin—CCTGAGCCTGTAGTTGACCGCTGCATAGCA
G4	**7**	TGCTATGCAGCGGTCAACTACAGGCTCAGGCTGGGTAAGGGTAAGGGTAAGGGTAA—Amine
G4	**8**	TTACCCTTACCCTTA

We monitored the formation of our SWCNT‐DNA‐QD heterostructures by casting diluted solutions on muscovite mica and imaging the substrate via atomic force microscopy (AFM). Figure 1b shows a representative image of SWCNT‐10 bp‐QD heterostructures (see also Figure S3, Supporting Information, for representative AFM images of the 20 and 30 bp hybrids; height analysis can be seen in Figures S3c and S4, Supporting Information).

The yields of formation of the SWCNT‐QD nanohybrids, as measured by AFM, were found to be ≈25%, where 100% of the heterostructures presented only one QD at one terminal end of the nanotube, i.e., were monofunctionalized.^19^ 2:1 SWCNT‐QD nanohybrids formation was minimized by the mildness of the aforementioned nanotubes acid treatment, so that the SWCNTs ends were not saturated with carboxylic defects.[[qv: 2b,17a,20]] Furthermore, it is unlikely that more than one QD would attach to the same end of the SWCNT, due to spatial constraints.[[qv: 12a]]

## 3 Photoluminescence

### 3.1 Solution‐Based Photoluminescence

**Figure 2:**
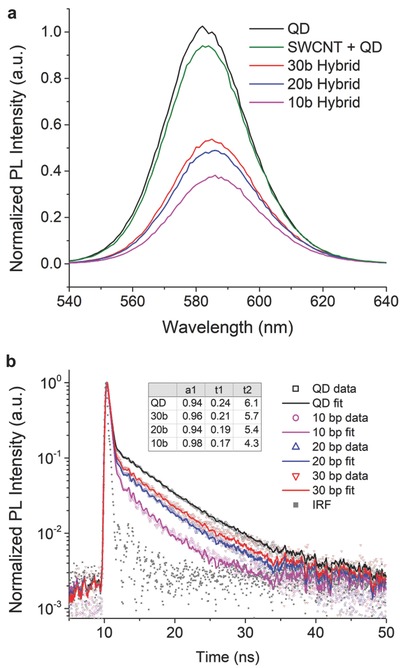
Photoluminescence spectra of pristine QDs and each SWCNT‐QD nanohybrid: a) SSPL spectra (as a control experiment, shown in green, SWCNTs underwent the same treatment as the hybrids but with no linker present) and b) time‐resolved PL spectra (the data were fitted with an iterative convolution of the instrument response function, IRF in gray, and a biexponential trace; the emission wavelength is 585 nm).

In order to probe the electronic coupling between the SWCNT and the QD in the monofunctionalized hybrids, PL investigations were carried out. All nanohybrids were prepared by keeping the concentration of QDs constant across all samples so as to accurately determine the communication with the CNTs. Steady‐state PL (SSPL) measurements showed that the QDs emission was progressively quenched to a higher degree as the nanoparticle approached the end of the SWCNT in our heterostructures. Specifically, the QDs in the 30 bp hybrids were quenched by 47% relative to the pristine QDs emission, while the QDs in the 20 and 10 bp hybrids were quenched 52% and 63% respectively (see **Figure** **2**). This suggests that the QDs are electronically coupled to the SWCNTs to a higher degree as they are positioned closer to the nanotubes.

Interestingly, SSPL also shows a small redshift (3–6 meV) of the emission peaks; the redshift increases as the linker length decreases. The effect is similar to that observed in thin films of electronically coupled QDs and can be attributed both to the change of the dielectric function surrounding each QD^21^ and increased electronic coupling between the QD and the CNT.^22^ The latter effect is likely predominant in our hybrid system since it is compatible with the increase of the quenching of the SSPL (as the linker length decreases) and can in principle lead to a delocalization of charge carriers and concomitant increase in the charge transfer (CT) effect.

Time‐resolved PL (TRPL) investigations were carried out in order to investigate the decay dynamics of the electronic excitations in our heterostructures and hence elicit the coupling mechanism between the two nanostructures forming the hybrids. Figure 2b shows that pristine QDs exhibit a lifetime decay with a biexponential trace, in agreement with observations made on similar QDs, with the shorter lifetime (*t*_1_) commonly attributed to the recombination of core states and the longer lifetime (*t*_2_) to radiative recombination of excitons involving surface states.[[qv: 11c,12a]] As the QD approaches the SWCNT in the monofunctionalized hybrids, both the lifetimes were observed to progressively shorten (see Figure 2b and inset table). At the same time, in the transition from 30 to 10 bp the emission follows a near‐monoexponential decay with up to 98% of the emission concentrated on the shorter lifetime, *t*_1_, that is attributed to the QDs core states. This in turn suggests that as the distance shortens, the surface states, attributed to *t*_2_, contribute more to the QD‐SWCNT coupling than the core states. Therefore, our results imply that, as the linker's length shortens, the electronic interaction between the QDs and the SWCNTs in the hybrids is dominated by a surface‐mediated CT process rather than by energy transfer (ET) losses.[[qv: 11c]]

Control experiments for both SSPL and TRPL were carried out, where only the DNA linkers were attached to the QDs, in the absence of the SWCNTs (see the Supporting Information). In the case of the SSPL (see Figure S5, Supporting Information), the observed quenching effect was not as significant as observed in the nanohybrids, and there was no significant difference in emission between each length of duplex. Moreover, TRPL showed no significant differences in lifetimes and amplitudes for the control QD‐DNA samples when compared to the pristine QDs (see Figure S6a and inset table, Supporting Information). Differently, by simply mixing QDs with SWCNTs in the absence of the DNA linkers, a shorter lifetime was observed compared to the pristine QDs, with a value comparable to the 30 bp nanohybrid (see Figure S6a,b, Supporting Information). This suggests that the average distance between the QD and the SWCNT in the mixture solution is comparable to the distance in the 30 bp hybrid.

In addition to monitoring the QD emission, SSPL studies were carried out on the SWCNTs for each nanohybrid as well as for the pristine nanotubes. The semiconducting SWCNTs used in these experiments emit, as expected, in the IR region (see Figure S7, Supporting Information). No significant difference in emission was observed between the nanotubes in the hybrids and the pristine SWCNTs. This is likely due to the high specificity of our functionalization, where single QDs are only tethered to the ends of the nanotubes, hence the electronic structure of the SWCNTs is preserved, with only local changes potentially occurring at the site of QDs attachment.

As noted previously, the SWCNTs were size separated to provide a narrower distribution of lengths, and the SWCNTs used in these measurements came from a range of fractions with average lengths from ≈330 to 130 nm. The changes observed in PL were consistent across all fractions, indicating that within the range used length did not have an impact on the quenching.

### 3.2 Single‐Molecule Photoluminescence

**Figure 3:**
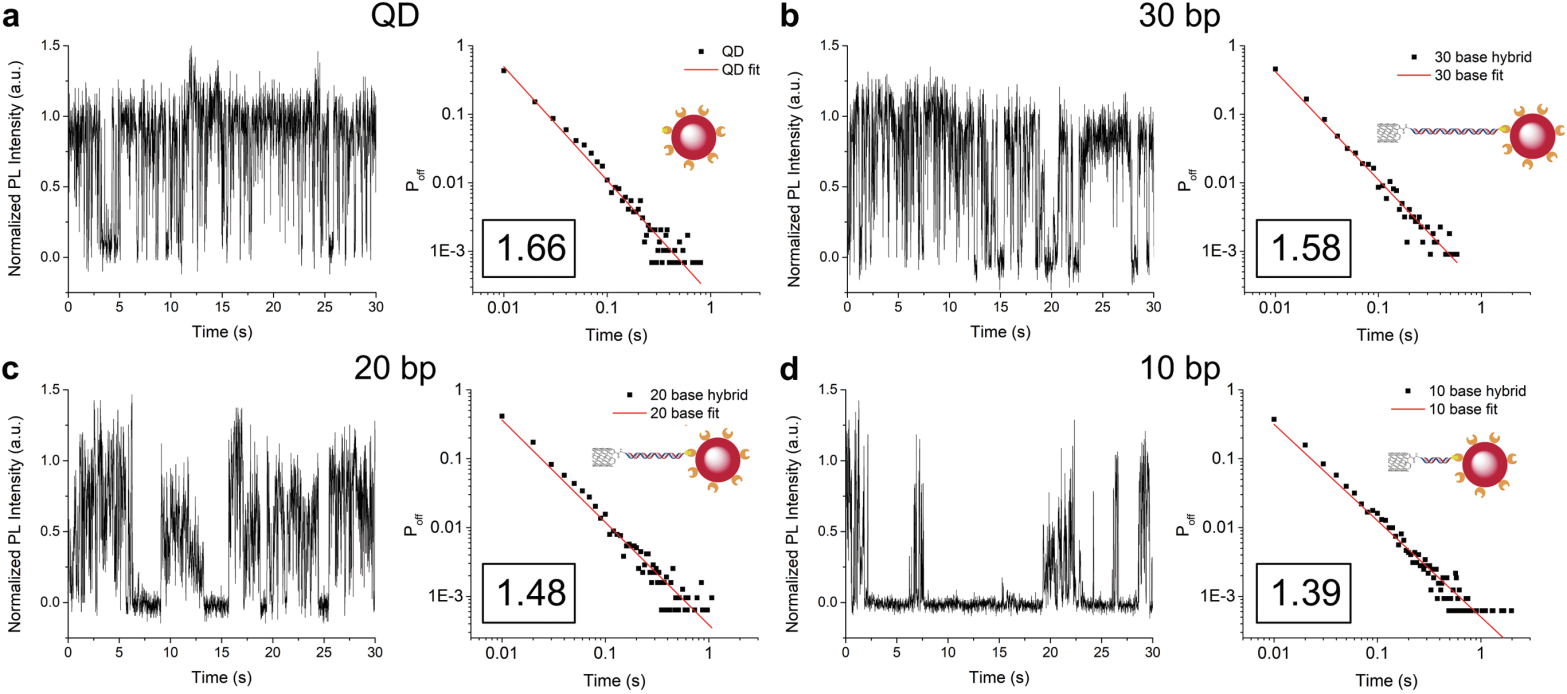
Representative intensity versus time plots and corresponding probability distributions (with exponent values inset) of single a) pristine QDs, b) 30 bp hybrids, c) 20 bp hybrids, and d) 10 bp hybrids.

To further monitor the electronic coupling with single‐particle resolution, single‐molecule measurements were performed. We cast low‐density films on glass substrates so to obtain physisorbed QDs or SWCNT‐QD hybrids spaced at least 1 µm apart, therefore optically resolvable (see the Experimental Section and Figure S8, Supporting Information). This allowed us to carry out PL studies of individual nanoparticles and heterostructures on surfaces. Emission intensities from single QDs and SWCNT‐QD hybrids were plotted against time; when progressing from pristine QDs to 30, 20, and 10 bp linked hybrids, longer “off” times (lower QD emissivity) were measured (see **Figure** **3** and Figure S9, Supporting Information). By accumulating the “off” time data for each set of heterostructures, probability distributions of off events can be constructed (see Figure 3 and the Supporting Information); these show a power‐law distribution.^23^ Pristine QDs were found to have an exponent of 1.66—in agreement with literature values[[qv: 12a,24]]—while the 30, 20, and 10 bp nanohybrids were seen to follow a decreasing trend with exponents of 1.58, 1.48, and 1.39, respectively. CNTs have shown to induce CT in QD‐CNT heterostructures,[[qv: 11c,12a,25]] by hampering exciton recombination in QDs, that are then left in a charged state for longer periods. The higher probability of “off” times we observe with decreasing distance between the tubes and the QDs in the hybrids can therefore be explained by the progressive increase of this CT as the dominant mechanism responsible for the observed electronic coupling:^26^ longer off periods imply longer charge state periods hence higher degree of CT. Additionally, it has been demonstrated that if ET is the (only) coupling mechanism no significant changes in QDs blinking are typically observed.[[qv: 12c]] These findings, obtained via single‐particle measurements, are in agreement with the aforementioned solution‐based SSPL and TRPL measurements shown in Figure 2. The observed trend confirms our ability to modulate the coupling between each component in our monofunctionalized SWCNT‐QD nanohybrids via the use of dsDNA as a linking moiety.

## 4 Stimuli‐Responsive Nanohybrids

**Figure 4:**
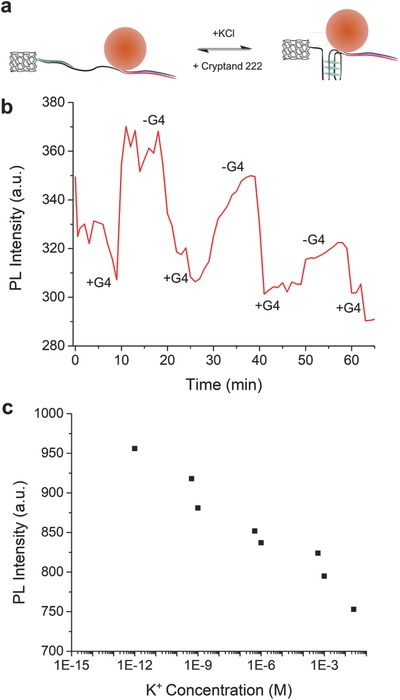
a) Scheme for the conformational changes of the SWCNT‐QD nanohybrid with G4 aptamer upon addition of K^+^ or cryptand 222; b) PL intensity plotted against time with alternating additions of K^+^ and cryptand 222. (+G4) indicates the formation of the G‐quadruplex, while (−G4) indicates the reversion to the linker's extended conformation; c) PL intensity plotted against the concentration of K^+^ indicating the range of sensitivity.

In order to further tune the assembly of heterostructures with dynamic control, we investigated the formation of reconfigurable 1:1 SWCNT‐QD nanohybrids. In particular, we designed a linker using a well‐known G‐rich sequence (TGCTATGCAGCGGTCAACTACAGGCTCAGGC**TGGGTAAGGGTAAGGGTAAGGGTAA**, where the bold lettering indicates the G4 forming portion) which forms a G4 in the presence of K^+^ cations (see the Supporting Information and Table 1). Upon addition of K^+^, the sequence folds into a G4 structure bringing the QD in closer proximity to the end of the SWCNT (see **Figure** **4**a and Figure S10, Supporting Information), therefore increasing the degree of coupling; this can be observed monitoring the quenching of the QDs' emission. Furthermore, the formation of the G4 is reversible: the addition of cryptand 222 (Figure S10c, Supporting Information) to the system and the resulting complexing with the K^+^ cations, restores the extended conformation of the G‐rich aptamer linker. Notably, by the sequential addition of K^+^ and cryptand 222, the conformation of the hybrid nanostructure could be cycled between contracted and extended as monitored by SSPL in real time (see Figure 4b and Figure S11, Supporting Information). With each cycle, the formation of the G4 loses efficiency, where after the third cycle the change in PL intensity is no longer clear. This is likely due to saturation of K^+^ salts and cryptand 222 in the solution.

Finally, we can notice that the quenching is concentration dependent, where a decrease in emission intensity was seen for K^+^ concentrations ranging from 1 pM to 25 × 10^−3^ m (see Figure 4c). While the G4 linker used here demonstrates the concentration‐dependent sensing of K^+^ cations (see also Figure S12, Supporting Information), this system may be assembled employing other stimuli‐responsive sequences selective to different target molecules or environmental conditions.

## 5 Conclusion

In conclusion, we presented, to the best of our knowledge, the first example of assembly with single‐molecule control of one‐to‐one heterostructures where the electronic coupling between an individual functional nanomoiety (a QD as proof of concept) and a carbon nanotube (acting as a potential nanoelectrode) was tuned with nanoscale precision both statically and dynamically via the use of DNA linkers of different length. Static, time‐resolved, and single‐molecule photoluminescence measurements demonstrated a distance‐dependent effect on the electronic coupling between the two nanomoieties in the hybrids; a closer proximity increased the extent of coupling. These measurements further indicated that CT becomes the dominating mechanism as the distance between the tubes and the QDs is controllably reduced. Additionally, we extended our studies to the assembly of reversibly reconfigurable 1:1 heterostructures employing a G‐rich DNA linker. This strategy allowed us to trigger the reconfiguration of the linker into a G4 structure—contracting the distance between the SWCNT and QD—by the addition of K^+^ and restore its initial extended conformation by adding cryptand 222 to the solution. This dynamic coupling was monitored in real time via SSPL, where the contracted heterostructures exhibited a quenched emission, while the emission was seen to recover in the extended hybrids. Moreover, the change in conformation was found to be sensitive to K^+^ concentrations from pM to mM, highlighting the additional sensing properties of the stimuli‐responsive platform developed.

By and large, the strategy we presented holds great interest for the fabrication of solution‐processable reconfigurable heterostructures with single‐molecule control, for optoelectronics, light harvesting, and sensing applications. The general applicability of this DNA‐programmed approach, beyond semiconductor nanocrystals which were here employed as a proof of concept, makes it of further relevance for the development of tunable, and stimuli‐responsive, single‐molecule systems based on the use of carbon nanoelectrodes.

## 6 Experimental Section

*SWCNT‐QD Nanohybrid Assembly*: SWCNTs (6.25 µg mL^−1^) were mixed in a 1:1 ratio with 2‐(*N*‐morpholino) ethanesulfonic acid (MES) buffer (0.2 m; pH 4.7; Thermo Scientific) containing 1‐ethyl‐3‐[3‐dimethylaminopropyl]carbodiimide hydrochloride (4 × 10^−3^ m; Sigma‐Aldrich) and *N*‐hydroxysulfosuccinimide (10 × 10^−3^ m; Sigma‐Aldrich). The solution was shaken at room temperature for 30 min, and then Dulbecco's phosphate buffered saline (DPBS), purchased from Thermo Scientific, was added in a 1:1 ratio. The dsDNA (see Table 1 for oligonucleotide sequences) was hybridized in DPBS at a concentration of 2.5 × 10^−6^ m. The dsDNA was added in a tenth of the total reaction volume giving a final dsDNA concentration of 250 × 10^−9^ m. The solution was shaken at room temperature overnight. Excess linker was removed by dialysis against tris(hydroxymethyl)aminomethane‐acetate‐ethylenediaminetetraacetic acid (TAE) buffer (1x) with NaCl (100 × 10^−3^ m) using Slide‐A‐Lyzer MINI Dialysis Devices with a 20 kDa cutoff purchased from Thermo Scientific. To conjugate QDs to SWCNTs (see Figure S2b, Supporting Information), a solution of Qdot 585 Streptavidin Conjugate (100 × 10^−9^ m) was added to freshly dialyzed SWCNT−DNA solution to give a final QD concentration of 5 × 10^−9^ m. The reaction was shaken for 40 h at room temperature in the absence of light. This concentration of QD was found to be optimum for maximizing the functionalization of SWCNTs while minimizing the number of free QDs. For the dynamic nanohybrids the DNA linker employed (see Table 1, sequences **7** and **8** were hybridized together to form a partial double strand adjacent to the amine modification) was attached to the SWCNTs in the same way as the linkers for the 10, 20, and 30 bp nanohybrids. The QDs were conjugated with sequence (**6**) found in Table 1 by mixing the QDs (100 × 10^−9^ m) and DNA (10 × 10^−6^ m) in TAE (1×) with NaCl (100 × 10^−3^ m). This solution was placed in a polymerase chain reaction (PCR) machine (Hybaid PCR Sprint) and heated to 47 °C, then cooled slowly at 0.1 °C per minute to room temperature. The QD‐DNA conjugates were filtered in Amicon Ultra‐0.5 mL Centrifugal Filters (100 kDa cutoff; purchased from Millipore) three times at 13000 RPM. QD‐DNA conjugates were mixed with the SWCNT‐DNA solution to give a final QD concentration of ≈5 × 10^−9^ m. The solution was then shaken at room temperature for 40 h in the absence of light. To contract or extend the distance between the QD and SWCNT in this nanohybrid, KCl (40 × 10^−3^ m) or Cryptand 222 (25 × 10^−3^ m) were respectively added: upon contraction of the structure due to the formation of the G quadruplex, sequence (**8**) dehybridized from sequence (**7**).

*Atomic Force Microscopy*: AFM was carried out on a Bruker Dimension Icon in PeakForce Tapping mode with ScanAsyst Air tips from Bruker. AFM was carried out on the SWCNT‐QD nanohybrids according to previously published methods.[[qv: 2b]] Briefly, freshly cleaved discs of muscovite mica (Agar Scientific) were cleaved with sticky tape, after which a solution of MgCl_2_ (Sigma‐Aldrich) was cast on the discs and blown dry with compressed air. The solutions of nanohybrids were deposited on the treated mica and incubated for 20 min on a shaker. Following incubation, the samples were rinsed with MilliQ water and blown dry with compressed air.

*Steady‐State Photoluminescence Spectroscopy*: The PL spectra of the QDs in prepared solutions of nanohybrids (as described above) were measured on an Agilent Cary Eclipse PL spectrometer. Samples were measured in a 45 µL volume Hellma fluorescence cuvette and an excitation of 405 nm was used. The SWCNTs emission spectra were recorded using the following experimental setup: A StradusTM diode laser (405 nm) modulated with a square wave using a function generator is used to excite the samples. The sample photoluminescence is then collected and collimated onto a Jobin Yvon Horiba Triax 550 spectrometer. A nitrogen cooled photomultiplier tube (PMT) (Photocool PC176TSCE005) is used to detect and multiply the signal collected from the spectrometer. A lock‐in amplifier, which is connected to a computer, is used to collect the emission intensity and record the spectra.

*Time‐Resolved Photoluminescence Spectroscopy*: Time resolved measurements were carried out on solutions of nanohybrids as described previously for SSPL. Samples were measured in a 45 µL volume Hellma fluorescence cuvette using an excitation wavelength of 460 nm and the emission decay was monitored at 585 nm. The following experimental setup was employed: Laser light is generated by the Continuum Surelite (SLI‐10) laser, the beam then passes through an optical parametric oscillator (Continuum Panther). Tuning the laser output at a specific wavelength is done using a computer controlled program. The beam passes through a series of lenses and it is focused onto the sample. The photoluminescence is then collected and collimated onto a Jobin Yvon Horiba Triax 550 spectrometer. A nitrogen cooled PMT (Photocool PC176TSCE005) is used to detect and multiply the signal collected from the spectrometer. The response from the PMT is then sent to an oscilloscope (LeCroy waverunner LT372). The photoluminescence spectra and lifetime data are recorded by a connected computer.

*Total Internal Reflection Fluorescence (TIRF) Microscopy*: Samples for TIRF microscopy were prepared using previously published methods.[[qv: 2b]] Glass coverslips were cleaned using piranha solution—a mixture of 3:1 sulfuric acid to hydrogen peroxide (30% w/w in water; Sigma‐Aldrich)—followed by a 10 min incubation in MilliQ water. The coverslips were then rinsed with ethanol and blown dry with compressed air. Nanohybrid and QD samples were cast using the same method for mica as previously described and were diluted before casting onto glass coverslips to ensure that the QDs were >1 µm apart, and hence optically resolvable at the single‐particle level (see Figure S8, Supporting Information). TIRF microscopy was carried out on an LSM 710 ELYRA PS.1 and the QDs were excited using a 488 nm laser. Time series of the nanohybrids were taken for 30 s with an exposure time of 10 ms. The probability distributions were analyzed using previously published methods.[[qv: 2b]] Emission intensity versus time plots for single QDs and nanohybrids were plotted using Image J, after which Origin and Matlab were used to determine the lengths of “off” times contained within a plot. Lengths of “off” times were accumulated and plotted in log–log space against frequency, which yielded linear probability distributions, from which the exponent value was calculated. See the Supporting Information for a more detailed description.

## Conflict of Interest

The authors declare no conflict of interest.

## Supporting information

Supplementary
