# Supplementary material for: Tuning the Coupling in Single‐Molecule Heterostructures: DNA‐Programmed and Reconfigurable Carbon Nanotube‐Based Nanohybrids
Source: Adv Sci (Weinh). 2018 Aug 14;5(10):1800596. doi: 10.1002/advs.201800596 (PMC6193148; doi:10.1002/advs.201800596)
Supplement: Supplementary file 1 — Supplementary [file ADVS-5-1800596-s001.pdf]

Copyright WILEY-VCH Verlag GmbH & Co. KGaA, 69469 Weinheim, Germany, 2018.

Supporting Information

for *Adv. Sci.,* DOI: 10.1002/advs.201800596

Tuning the Coupling in Single-Molecule Heterostructures: DNA-Programmed and Reconfigurable Carbon Nanotube-Based Nanohybrids

*Mark Freeley, Antonio Attanzio, Alessandro Cecconello, Giuseppe Amoroso, Pierrick Clement, Gustavo Fernandez, Felice Gesuele, and Matteo Palma**

Copyright WILEY-VCH Verlag GmbH & Co. KGaA, 69469 Weinheim, Germany, 2018.

Supporting Information

Tuning the Coupling in Single-Molecule Heterostructures: DNA-Programmed and Reconfigurable Carbon Nanotube-Based Nanohybrids

*Mark Freeley, Antonio Attanzio, Alessandro Cecconello, Giuseppe Amoroso, Pierrick Clement, Gustavo Fernandez, Felice Gesuele, and Matteo Palma**

SWCNT Preparation

Oxidized SWCNTs were prepared by chemical oxidation to cut SWNTs into open-ended tubes.[1] For the experiment, 8 mg HiPco nanotubes (Carbon Nanotechnologies Inc.) were suspended in 10 mL of a 3:1 mixture of concentrated H_2SO_4 (95-98 wt %; Sigma-Aldrich)/HNO_3 (69 wt %; VWR) and sonicated in a water bath for 15 min at 30 °C. Then, the SWCNTs suspension was collected on a 0.1 µm centrifugal filters (Merck Millipore Ltd.), washed with DI water and allowed to dry. The acidic cutting step opens the ends of the SWNTs and introduces carboxyl groups at the open-ends and at defect-sites of the SWCNTs, which represent useful sites for further functionalization.

Furthermore, the oxidized SWCNTs were dispersed in DI water according to published procedures.[2] Briefly, 1 mg of oxidized SWCNTs was suspended in an aqueous solution, containing NaCl (0.1 M; Fisher Scientific) and $d(GT)_{20}$-ssDNA (Integrated DNA Technologies). The suspension was kept in an ice-water bath and sonicated (Ultrasonic Cleaner, VWR) for 90 minutes. After sonication, the dispersion was divided into 0.1 ml aliquots, centrifuged for 90 minutes at 13000 RPM to remove insoluble materials, and the supernatant collected. The dispersion process not only allows the dispersion of SWCNTs in water, but also protects the side-wall of nanotubes leaving the open-ends available for a direct functionalization.

At the end, to obtain a length separation for tube fractions, DNA-wrapped SWCNTs were separated by means of Size-Exclusion Chromatography (SEC).[3] A 1.5 mL seat capillary (Agilent Technologies) and three columns (Sepax technologies, Inc) connected in series, with pore size of 2000 Å, 1000 Å and 300 Å respectively, were mounted on a HPLC system (Agilent 1100 Series). The functional groups on the stationary phase, 5 µm silica beads, are negatively charged to prevent the adsorption by the phosphate groups of DNA. In a typical run, 300 µL of DNA-wrapped SWCNTs solution were eluted with a buffer solution (pH 8), containing 3-(N-morpholino)propanesulfonic acid (MOPS; 10 mM; Sigma-Aldrich) and NaCl (0.2 M), at a flow rate of 0.25 mL/min. Elution was collected from min 33 to min 44 retention time at 0.25 mL/ fraction (see Figure S1). The peak at ~50 min retention time is identified as free DNA. Therefore, with SEC it is possible to separate SWCNTs by length and also to purify the DNA-wrapped SWCNTs from the free DNA in solution. The tube fractions mainly used in this work are 40 and 41. To quantify the average length in the fractions, we randomly picked 100 tubes from the AFM images of each fraction and measured their lengths. A statistical analysis resulted in 154.5 ± 78.5 nm and 130.2 ± 61.1 nm tube length for fractions 40 and 41, respectively.

Statistical Analysis of Blinking

Emission intensity was plotted as a function of time for single QDs and nanohybrids using Image J, after which Origin and Matlab were used to determine the lengths of “off” times contained within a plot. At least 25 QDs were analyzed per sample. A threshold intensity was set for each sample in order to identify an “on” or “off” state by taking the average intensity of the background and adding 3 times the standard deviation. Lengths of “off” times were accumulated and plotted in log-log space against frequency, which yielded linear probability distributions, from which the exponent value was calculated.

The “off” times were binned into a histogram with a 10 ms bin width. The “off” times from each hybrid were accumulated and treated as an ensemble. Probability distributions of “off” times are described by equation 1, where $P^{off}(t_i)$ is the normalized probability of “off” states for a duration time of t_i. This is given by $N(t_i)$, the number of “off” events during the bin time t_i, divided by the sum of all “off” events in a histogram.

$$P^{off}(t_i) = \frac{N(t_i)}{\sum_{i=1}^{n} N(t_i)} \quad (1)$$

The frequency of “off” times is then plotted against the binning time in log-log space (see Figure 3). This can be fit with a power law model given by equation 2, where the slope, m, is equivalent to the exponent, t is the bin length, and $P^{off}(t)$ is the analytical function equivalent to equation 1.

$$P^{off}(t) = bt^{-m} \quad (2)$$

Table S2 contains the data for each probability distribution.

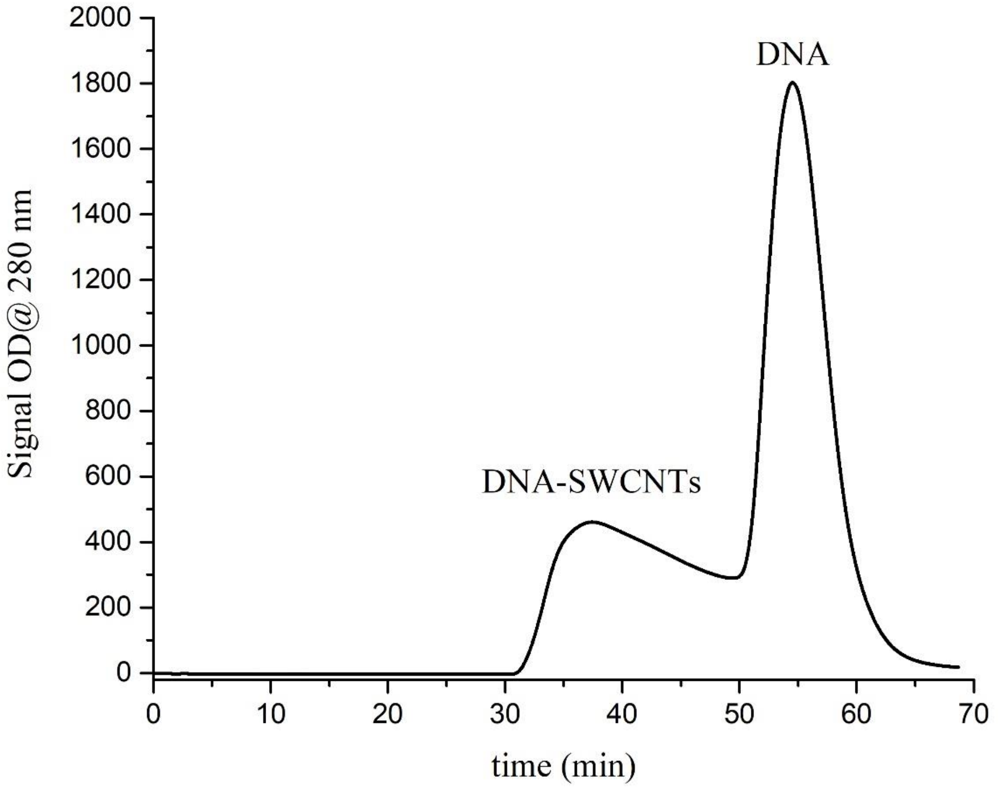

Figure S1. Chromatogram of size-exclusion column separation of DNA-wrapped SWCNTs.

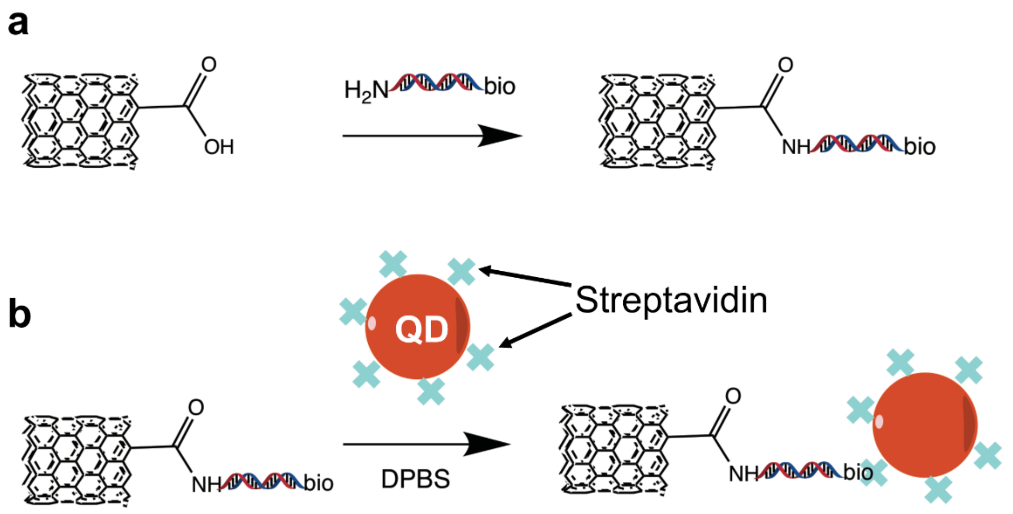

Figure S2. Scheme of assembly of the SWCNT-QD nanohybrids. (a) Functionalisation of SWCNTs with dsDNA. (b) Attachment of QDs to SWCNT-DNA conjugates.

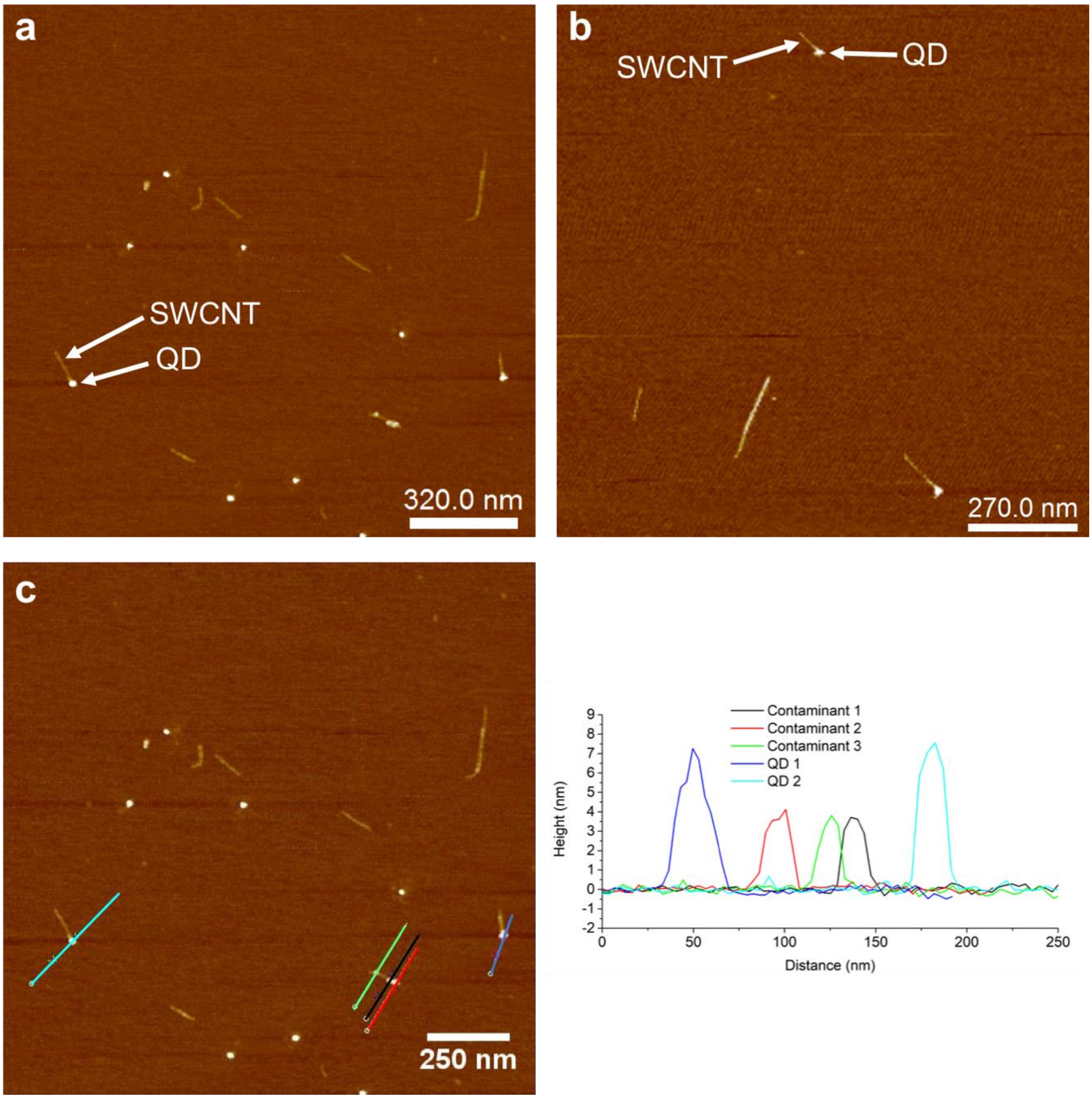

Figure S3. Representative AFM images of the (a) 20 bp and (b) 30 bp nanohybrids on mica. SWCNTs functionalized with QDs can be distinguished as demonstrated in (c), which shows height analysis of the AFM image (a) where the heights of QDs and adsorbates or contaminants are compared, with QDs exhibiting a height of ~7 nm, while the adsorbates exhibit heights of ~3.5 – 4 nm.

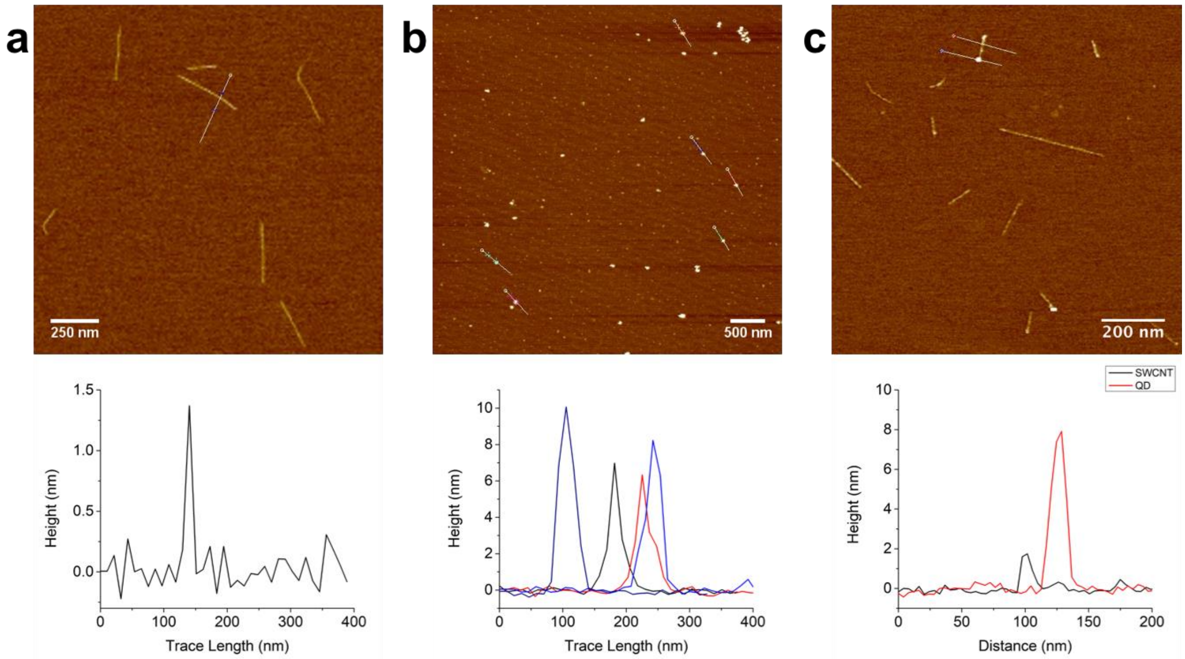

Figure S4. AFM height analysis of (a) a SWCNT, (b) QDs, and (c) a 10 bp nanohybrid.

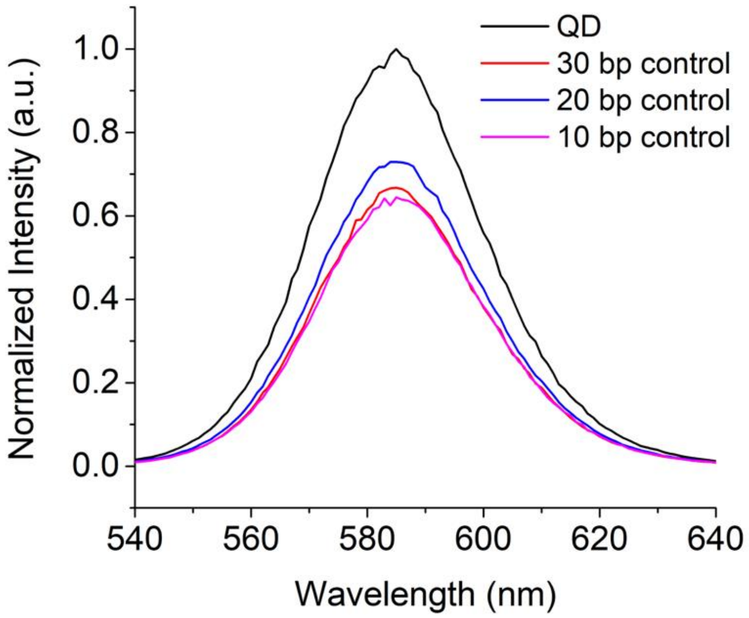

Figure S5. Control experiments for SSPL where each length of dsDNA linker was conjugated to QDs in a one-to-one ratio.

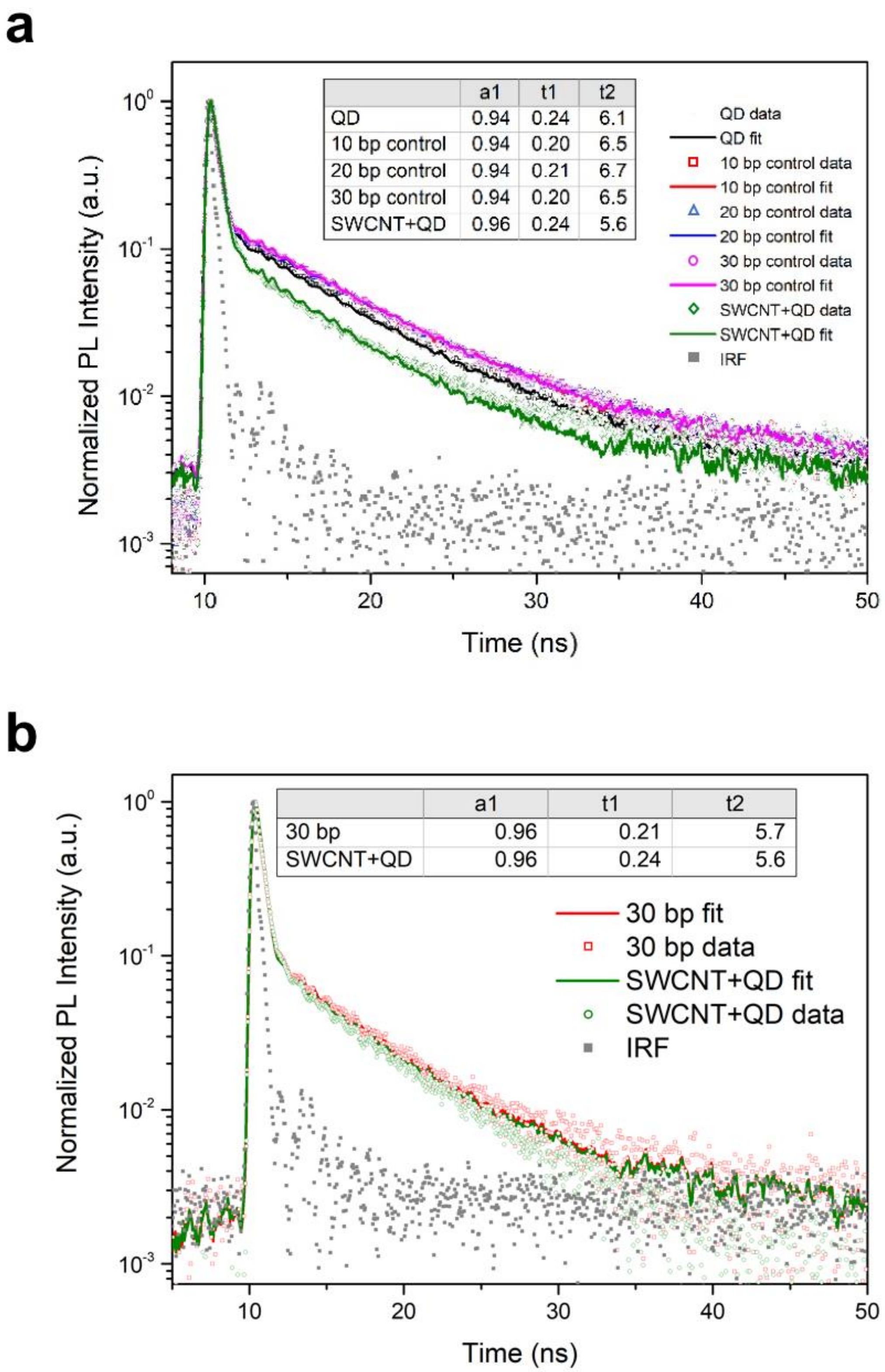

Figure S6. Control experiments for TRPL where (a) dsDNA linkers were conjugated to QDs in a one-to-one ratio, and where (b) dialyzed pristine DNA-wrapped SWCNTs were mixed with QDs in the same ratios as the nanohybrids, i.e. 5 nM of QDs.

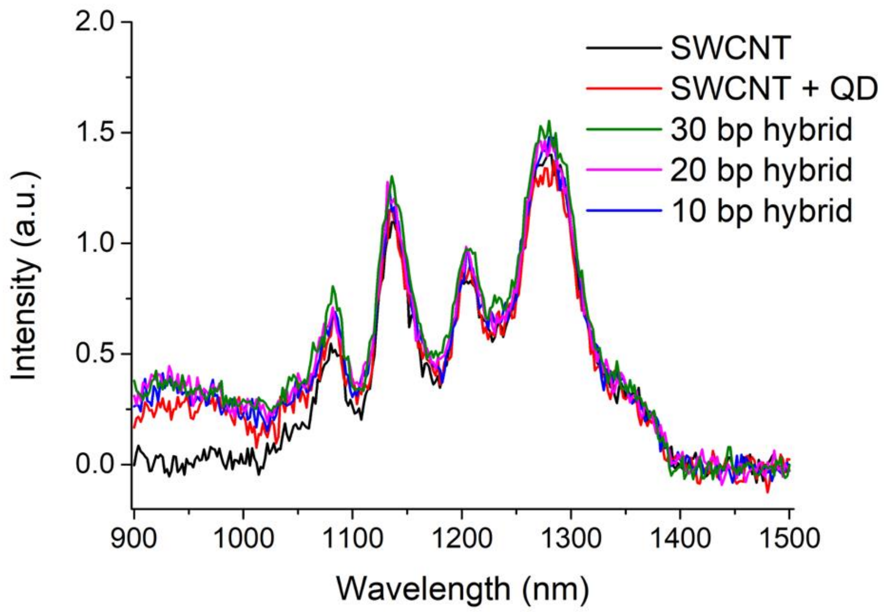

Figure S7. SSPL spectra for the pristine SWCNTs, mixture, and each nanohybrid. Each peak corresponds to a different chirality of SWCNT, as there is a mixture of chiralities in the SWCNT solution. No significant difference can be seen among all the traces, nor between pristine SWCNTs and hybrids. Considering that only single QDs are attached at the ends of the nanotubes, variations in the electronic structure of the SWCNTs are not expected, with only local changes potentially occurring at the area of QD attachment.

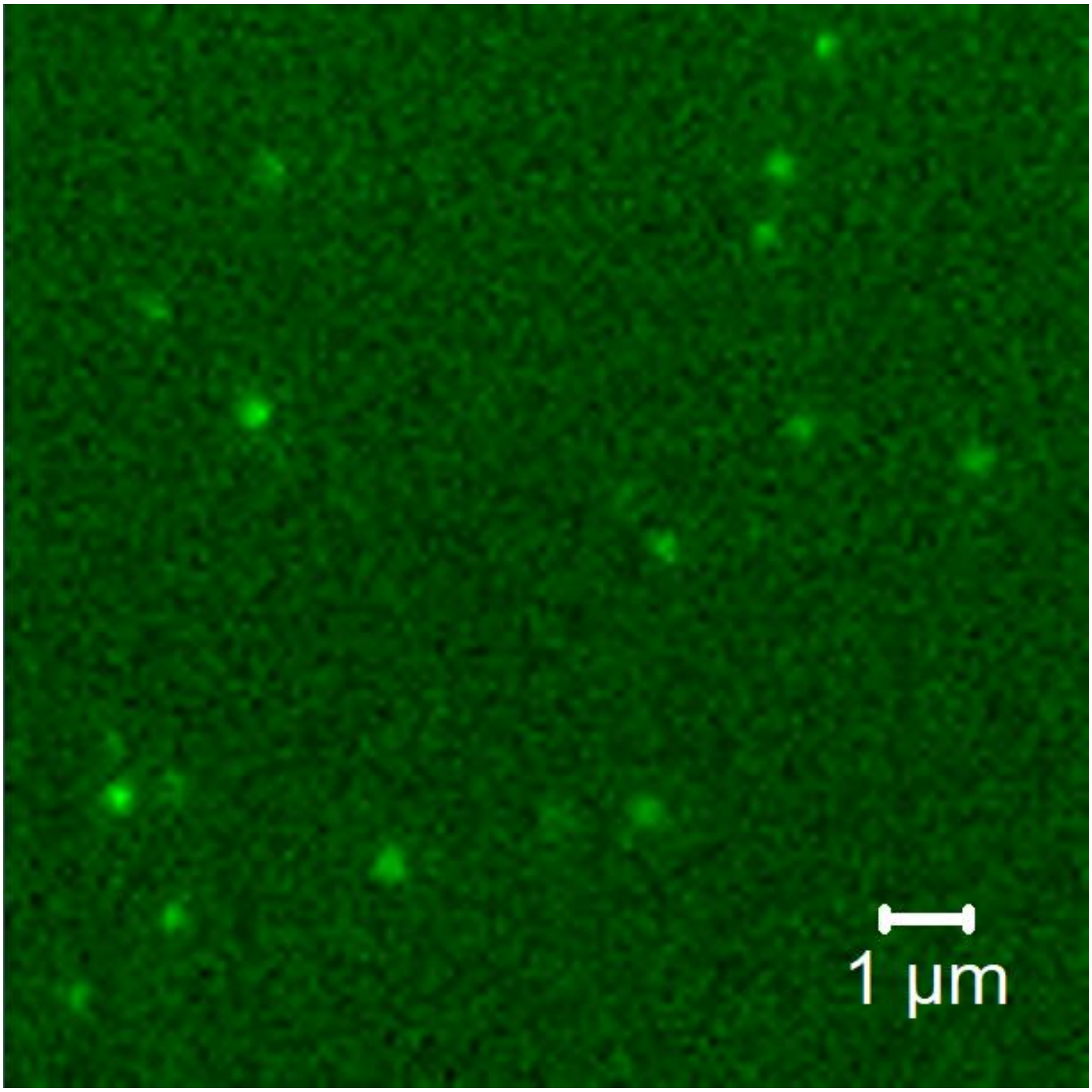

Figure S8. Representative TIRF microscopy image of a SWCNT-QD sample. Each green dot shows a single QD.

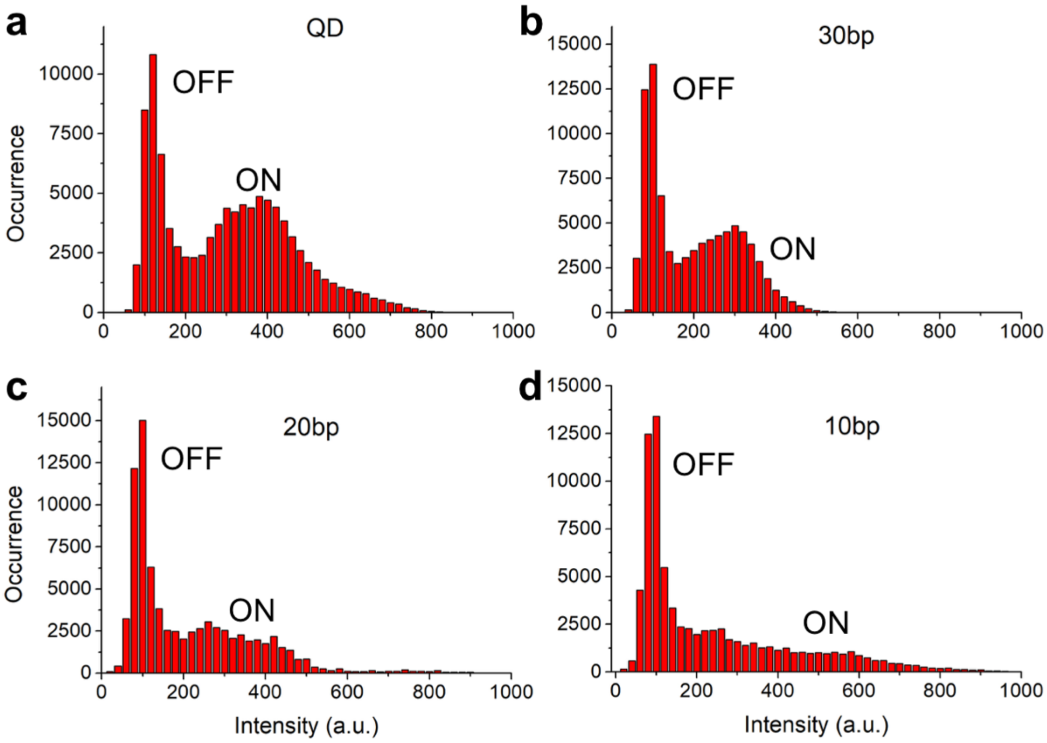

Figure S9. Histograms of the emission intensities from accumulated blinking traces for (a) pristine QDs, (b) 30 bp hybrids, (c) 20 bp hybrids, and (d) 10 bp hybrids. The areas of the distribution of "on" states for the 30 bp, 20 bp, and 10 bp nanohybrids are 20%, 30%, and 38% less than the area for the "on" states of the pristine QDs.

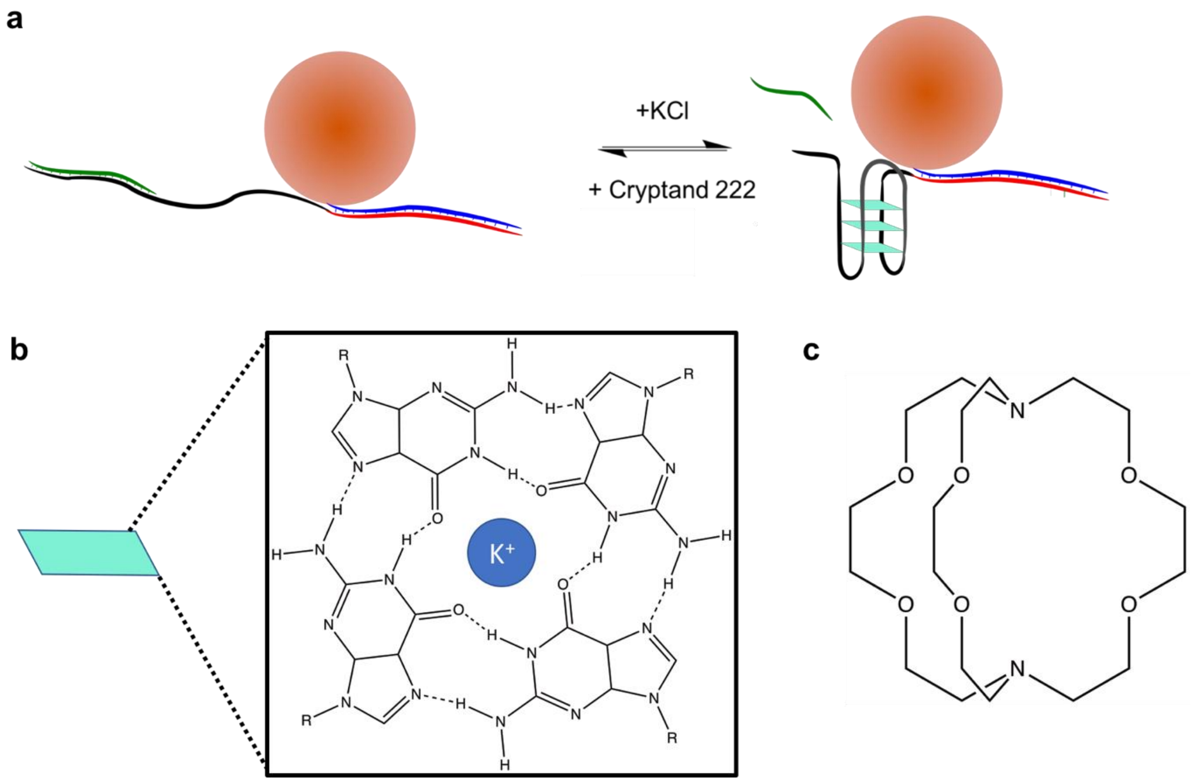

Figure S10. (a) Scheme for the G-quadruplex formation on the SWCNT-QD nanohybrids. When K^+ is added to the system, a G-quadruplex is formed by means of stacked G-quartets (light blue parallelograms, where each corner represents a guanine). The G-quadruplex formation releases sequence **8** (green). When cryptand 222 is added the K^+ is removed from the system and sequence **8** rehybridises, reverting the system to the extended conformation. (b) The structure of the G-quartet. (c) The structure of cryptand 222

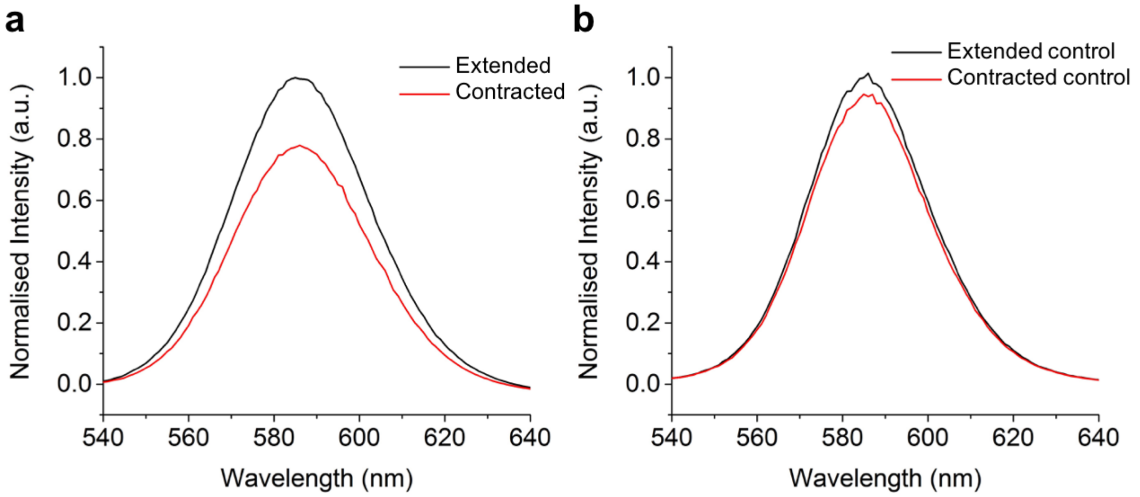

Figure S11. SSPL for (a) the G4 linked nanohybrid in extended (black line) and contracted (red line) as well as (b) a control experiment where only the G4 sequence was hybridized to the QD, with no SWCNT.

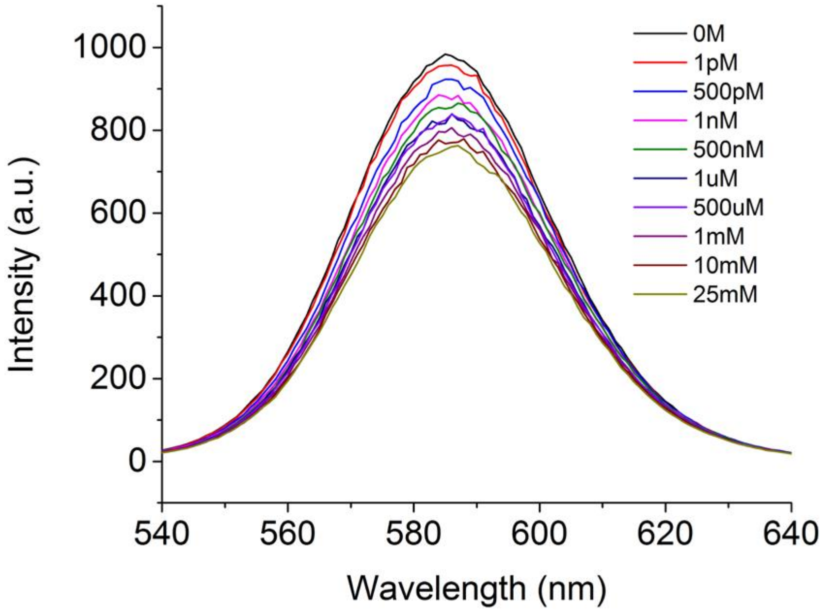

Figure S12. SSPL of the G4 linked nanohybrids, where K^+ was titrated from 1 pM to 25 mM.

References

[1] J. Zhang, H. Zou, Q. Qing, Y. Yang, Q. Li, Z. Liu, X. Guo, Z. Du, *J Phys Chem B* **2003**, 107, 3712.

[2] M. Zheng, A. Jagota, E. D. Semke, B. A. Diner, R. S. McLean, S. R. Lustig, R. E. Richardson, N. G. Tassi, *Nat Mater* **2003**, 2, 338.

[3] X. Huang, R. S. McLean, M. Zheng, *Anal Chem* **2005**, 77, 6225.
